# Can extreme rainfall associated with landslides affect air quality? A case study in a coastal city (São Sebastião, São Paulo State, Brazil)

**DOI:** 10.1007/s10661-026-16071-w

**Published:** 2026-10-22

**Authors:** Leticia Rocha Magalhães, Simone Andréa Pozza, Danilo Covaes Nogarotto

**Affiliations:** Faculdade de Tecnologia, Universidade Estadual de Campinas (UNICAMP), Limeira, São Paulo, Brazil

**Keywords:** Particulate matter, Air pollution, Extreme precipitation events, Cluster analysis

## Abstract

This study investigated the impact of an extreme precipitation event and the subsequent landslides that occurred in São Sebastião municipality (São Paulo State, Brazil) in February 2023 on particulate matter (PM_10_ and PM_2.5_) concentration. Hourly air quality and meteorological data from December 2021 to February 2025 were analyzed. The methodology combined cluster analysis (CA) using the *k*-means algorithm to classify meteorological patterns and the Openair package for spatio-temporal assessment. CA identified a specific group of days associated with anomalous behavior during the extreme event. Analyses using bivariate polar plots and variation in time plots revealed that, while intense rainfall initially promoted wet scavenging of pollutants, the post-landslide period was characterized by a 124% increase in PM_10_ concentrations and a 93.3% increase in PM_2.5_ concentrations. Two additional high precipitation events were examined for comparison, showing that PM also decreased during intense rainfall, while the post-rainfall recovery differed among events. These results indicate that the February 2023 increase cannot be explained by landslide occurrence alone and may have reflected the combined influence of meteorological conditions, changes in local activities, and potential particle resuspension*.* Overall, the findings highlight the importance of adequate monitoring networks in place for understanding the secondary environmental effects of extreme rainfall and disaster events in vulnerable coastal areas.

## Introduction

Approximately 99% of the world’s population is exposed to fine particulate matter (PM) at concentrations exceeding the limit established by the World Health Organization (WHO, 2023) . According to Brauer et al. (2024), air pollution is one of the main global risk factors, ranking fourth in terms of its contribution to premature mortality, and remains a major driver of disability-adjusted life years (DALYs) worldwide.

Of the main air pollutants, particulate matter stands out. This consists of dust, smoke, and other solid or liquid substances that are suspended in the air. Based on its aerodynamic diameter, it is commonly classified as PM_2.5_ (less than 2.5 µm) and PM_10_ (less than 10 µm). Exposure to PM_2.5_ poses a greater health risk, as these fine particles can penetrate deep into the respiratory system and reach the pulmonary alveoli (Sangkham et al., 2024). PM can originate from both primary and secondary sources. Primary particles are emitted directly from sources such as vehicles, industrial activities, and the resuspension of soil dust, whereas secondary particles are formed through atmospheric reactions involving gaseous precursors, including formation of secondary organic and inorganic aerosols (Lee et al., 2023). Furthermore, when high concentrations of pollutants occur over a short period, generally due to meteorological conditions that hinder dispersion, it is called a critical air pollution episode (Morawska et al., 2021).

An average seasonal pattern in PM_2.5_ concentration is observed, with higher levels in winter and lower levels in summer. This variation is associated with the typical meteorological factors of each season, such as temperature and precipitation. In coastal regions, however, these variations tend to be less pronounced due to more stable climatic conditions, which are influenced by the proximity to the sea (Dhital, 2024). In this context, precipitation is one of the main agents controlling air quality through the mechanism of wet deposition. According to Wang et al. (2023), rainfall effectively cleans the atmosphere, immediately reducing PM concentrations in proportion to the intensity and duration of the precipitation event.

The municipality of São Sebastião, located on the coast of São Paulo State, experienced an extreme rainfall event in the early hours of February 19, 2023. The total precipitation that day reached 680 mm. The landslides followed blocked roads, disrupted water and food supplies, and resulted in fatalities and widespread displacement (G1, 2024). The event was associated with intense rainfall linked to a stationary cold front, which was further intensified by orographic effects from the Serra do Mar Mountain range (Alcântara et al., 2025b).

A landslide strips away the vegetation that normally holds soil in place, leaving it exposed and loose. This phenomenon is closely factors that Alcântara et al. (2025b) identified as the most important for landslide risk in São Sebastião: slope, soil moisture, and terrain dissection. Once the soil is exposed, it becomes a potential source of PM. McMullen et al. (2021) showed that particle concentrations can remain elevated for up to 20 h after rainfall, as deposited material gets resuspended and redistributed into the atmosphere. After the February 2023 event, 983 landslide areas were mapped in São Sebastião (Coelho et al., 2024). This suggests that a considerable amount of freshly exposed soil could have plausibly added to PM levels in the following days.

However, Coelho et al. (2024) did not analyze the landslides in terms of air quality. The present study aims to investigate the effects of this extreme event on air quality, particularly PM concentrations, and to assess the potential role of soil resuspension caused by landslides as a contributing factor to the observed atmospheric variations.

## Methodology

### Study area

Considered the oldest municipality on São Paulo’s North Coast (Prefeitura Municipal de São Sebastião, 2021), it is located at 23°45′36″ S and 45°24′35″ W and covers a total area of 402.39 km^2^, 30.98 km^2^ of which is urbanized. Situated within the Atlantic Forest biome, the city is estimated to have a population of 84,019 inhabitants for 2024, with a demographic density of 202.77 inhabitants per km^2^, according to the Brazilian Institute of Geography and Statistics (IBGE, 2026).

**Fig. 1:**
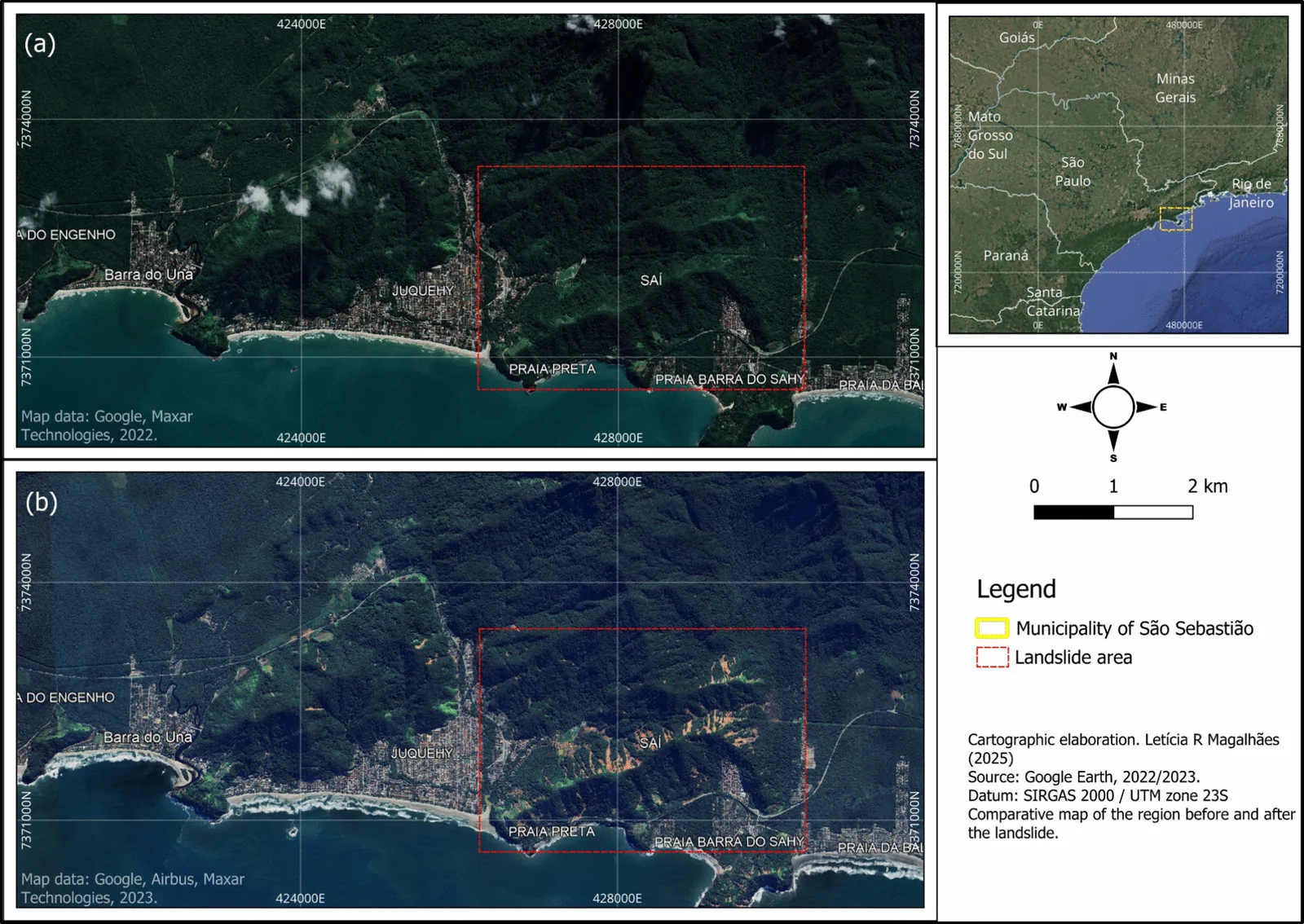
Orbital images from Google Earth Platform of the conditions **a** in May 2022 before the event and **b** in June 2023 after the event in the Vila Sahy region, São Sebastião

The local topography features altitudes ranging from sea level to over 1000 m. The Serra do Mar mountain range extends along the coastline and contributes significantly to the occurrence of intense rainfall in the region by orographic mechanisms. The municipality has a tropical rainforest climate, with high rainfall, typically concentrated between November and March (Alcântara et al., 2025b). Figure 1 shows that the highest incidence of landslides in February 2023 occurred in Vila Sahy. The orbital images from Google Earth Platform in Fig. 1 were selected for their superior spatial resolution and cloud-free coverage among the most recent available data to ensure greater clarity in identifying the affected areas.

São Sebastião experiences substantial seasonal increases in vehicular traffic, particularly during holidays and the summer tourist season. The SP-055 (Rio-Santos Highway), the main coastal road crossing the municipality, is subject to intense traffic and congestion during these periods due to the large influx of visitors to the northern coast of São Paulo. According to traffic estimates published by the São Paulo Department of Highways (DER-SP, 2023), the SP-055 can reach hundreds of thousands of vehicles during holiday periods.

It is an important coastal tourist destination that also hosts significant logistical, port, maritime, and petroleum-related activities. The local economy is strongly associated with tourism, port logistics, oil and gas activities, offshore services, and commerce, while the Port of São Sebastião operates as a multipurpose port facility, with areas for cargo handling, berthing infrastructure, and internal road circulation (Companhia Docas de São Sebastião, 2024).

São Sebastião generally presents good air quality with respect to the PM. According to the Environmental Company of the State of São Paulo (CETESB, 2026), in 2025, 97.3% of the valid 24-h PM_10_ measurements were classified as good (0–50 µg/m^3^), while moderate air quality conditions (> 50–100 µg/m^3^) were observed during the remaining 2.7%. For PM_2.5_ concentrations all valid 24-h measurements were classified as good (0–25 µg/m^3^) throughout the entire year. In the same period, the mean PM_2.5_/PM_10_ ratio was approximately 0.4, indicating that fine particles accounted for about 40% of the PM_10_ mass, while the remaining fraction was mainly associated with particles between 2.5 and 10 µg. This indicates an important contribution of the coarse particle fraction to ambient PM_10_ concentration in the municipality.

### Data collection

**Fig. 2:**
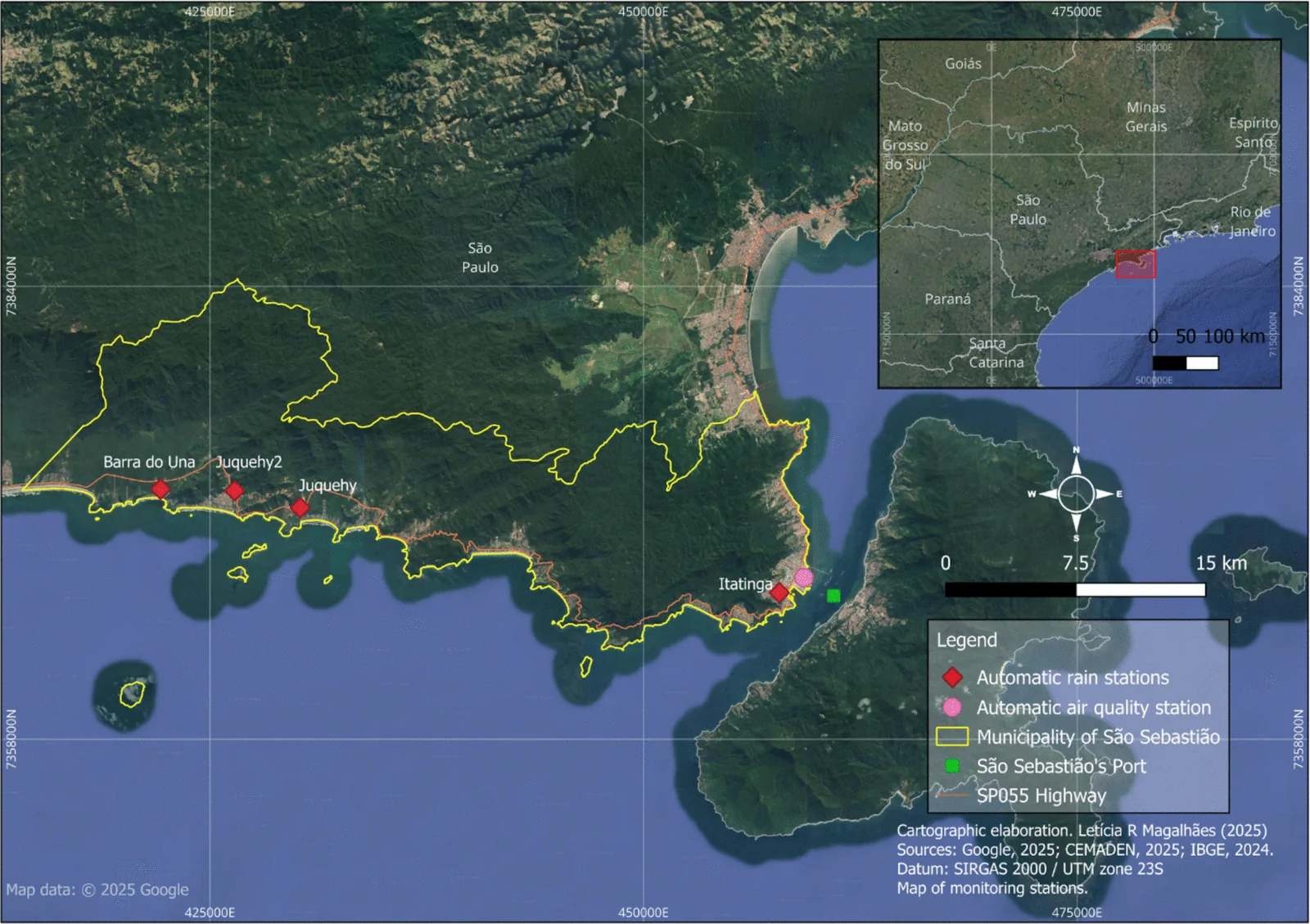
Location of the selected rain stations and air quality stations in São Sebastião

In the state of São Paulo, air quality monitoring is conducted by the Environmental Company of the State of São Paulo (CETESB, 2026). Hourly concentration data (in µg/m^3^) for the studied pollutants (PM_2.5_ and PM_10_) were obtained from the air quality platform (QUALAR, 2025). Additionally, meteorological variables, such as temperature, relative humidity, wind speed, and wind direction, were obtained from the same platform, using data from the municipality’s sole automatic monitoring station (Fig. 2). The station is located at 23° 48′ 22″ S 45° 23′ 54″ W and has been operational since November 2019. However, valid PM data are only available from December 2021. Consequently, this study covers the period from December 2021 to February 2025.

Air quality monitoring in Brazil is guided at the national level by the National Air Quality Standards (NAQS), which were established by the National Environmental Council through CONAMA Resolution No. 506/2024 (Brasil, 2024). State-level regulations also apply, such as São Pauloʼs Decree No. 59.113/2013 (São Paulo, 2013). The NAQS are organized into progressive stages, consisting of four intermediate standards that gradually advance toward a stricter final standard aligned with the WHO guidelines. This phased approach gives municipalities time to comply gradually with the NAQS’s increasingly stringent criteria. The decree also defines daily NAQS (24-h periods) and annual NAQS (calculated as an annual arithmetic mean). This study considers first standards (valid until December 31, 2024) and second standards (valid from January 1, 2025, to December 31, 2032), which cover the 2023–2025 analysis period.

Rainfall data were obtained from the National Center for Monitoring and Early Warning of Natural Disasters (CEMADEN, 2025) via four rain stations out of the 18 available in the study area. These stations record measurements every 10 min during precipitation events and every hour during dry periods. For the Vila Sahy analysis, the three stations were initially selected for their spatial representativeness of the rainfall associated with the event: Barra do Una, Juquehy, and Juquehy2 (Fig. 2). Additionally, the Itatinga station was included because it is the closest to the CETEB air quality monitoring station. The latter is located approximately 30 km from the event epicenter. The coordinates for the four stations used in this study are listed in Table 1.

**Table 1:** Geographic coordinates of the rain stations in São Sebastião used in this study

Station’s name	CEMADEN’s code	Coordinates	
		**Latitude**	**Longitude**
Barra do Una	355070402A	23° 45′ 28″ S	45° 45′ 50″ W
Juquehy	355070410A	23° 46′ 04″ S	45° 41′ 05″ W
Juquehy2	355070411A	23° 45′ 32″ S	45° 43′ 19″ W
Itatinga	355070409A	23° 48′ 46″ S	45° 24′ 50″ W

### Statistical analysis

All analyses in this study were performed using R software (R Core Team, 2025), version 4.5.1. Cluster analysis (CA) was used to investigate the dataset’s internal structure by grouping observations based on their similarity. In this analysis, observations with similar characteristics were grouped into the same cluster while maintaining differences between groups. CA has become an important tool in atmospheric and air pollution studies, particularly for investigating climate and meteorological data (Govender & Sivakumar, 2020).

For the CA application, hourly data from the QUALAR and CEMADEN platforms, covering the period from December 1, 2021, to February 28, 2025, were converted into daily data. In the present study, CA was used to group days with similar meteorological behavior and identify the main atmospheric patterns during the analyzed period. Only the following meteorological variables were included in this stage of the analysis: precipitation, temperature, relative humidity, wind speed, and wind direction. The analysis included only days with complete records of all the selected variables. This resulted in 849 valid days, equivalent to 71.59% of the total study period. PM concentrations were excluded at this point so that the meteorological conditions associated with the pollutant dispersion and accumulation could be evaluated separately.

A comparative analysis of the *k*-means and Ward methods was conducted to determine the most suitable approach for this study. Both methods used the Euclidean distance as the similarity measure, considering the nearest-neighbor criterion (Govender & Sivakumar, 2020). Also, the optimal number of clusters was evaluated using the Silhouette coefficient and Elbow methods.

The *k*-means method performed better in evaluating meteorological regimes and atmospheric variables variability in São Sebastião. The Ward method produced larger and more homogeneous groups with some overlap, whereas the *k*-means method identified clusters with more distinct patterns.

Based on these results, the *k*-means method was chosen for the analysis. This non-hierarchical clustering technique is widely used due to its simplicity and efficiency (Bano et al., 2024). The *k*-means algorithm requires the number of clusters (*k*) to be defined prior to analysis. It works by distributing the data into groups and adjusting the centroid positions until the clusters stabilize (Miraftabzadeh et al., 2023). This method is suitable for large datasets, though it is sensitive to the initial choice of k.

During the clustering phase, three clusters were defined using the k-means method. One of these groups, Cluster 3, contained the days of the event, representing the most critical meteorological condition. This set of days was defined as the “control group.” The days in the control group were divided into two categories: Days of the Event (DE), which corresponded to February 18 and 19, 2023, and were associated with the extreme event analyzed in this study; and Remaining Days (RD), which included the other days classified in the same cluster.

### Openair

The Openair package, developed for the R statistical environment, is an essential tool for analyzing air quality data. It provides functions for data processing, modeling, and visualizing atmospheric time series, combining meteorological information to help identify temporal and spatial patterns of pollutants. This study used several tools, including polarPlot function, which was applied to relate pollutant concentrations to wind direction and speed, while the windRose function was used to characterize the frequency distribution of wind direction and speed. The timePlot function was used to examine the hourly evolution of PM_10_ and PM_2.5_ together with wind vectors, allowing short-term changes during and after the event to be visualized. The timeVariation function was used to assess hourly, weekly, and seasonal pollutant patterns; and the theilSen function trend analysis, which was applied to evaluate significant temporal variations and directional patterns in PM concentrations (Carslaw, 2019; Carslaw & Ropkins, 2012).

This study used Openair to analyze the PM_10_ and PM_2.5_ time series, focusing on variations associated with precipitation, particularly during the February 2023 extreme event. These functions enable us to evaluate how meteorological factors influenced air quality before and after the landslides. They also helped us identify patterns in PM behavior in the region.

Polar plots were generated using the polarPlot function and non-parametric weighted regression (NWR) to estimate the average PM_10_ and PM_2.5_ concentrations according to wind direction and wind speed. To conduct a more detailed analysis, the hourly data were divided into three periods, considering a 2-day lag before and after the event: pre-event (February 16–17, 2023), during the event (February 18–19, 2023), and post-event (February 20–21, 2023). Only valid wind speed records (> 0 m/s) were considered to ensure consistency in the analysis.

To complement the polarPlot analysis and provide a more detailed characterization of post-event wind conditions associated with PM concentrations, the windRose function was applied using hourly wind speed, and wind direction data (Carslaw, 2019; Carslaw & Ropkins, 2012). The analysis focused on February 20–22, 2023, which corresponded to the period with the highest post-event PM concentrations. This approach made it possible to identify the wind sector and speed ranges most frequently associated with PM concentrations during the post-event period.

The timePlot function was used to examine the hourly evolution of PM_10_ and PM_2.5_ from February 18 to 22, 2023, together with wind vectors representing wind direction and relative wind speed. This analysis provided a more detailed view of short-term changes in PM concentrations during and after the event (Carslaw, 2019; Carslaw & Ropkins, 2012).

A temporal analysis of PM was performed using the timeVariation function, focusing on the week of the event, between February 13 and 19, 2023, which included a 5-day pre-event lag period. To facilitate comparison, a reference period was established using PM data from every February between 2022 and 2025. To avoid duplication of data, corresponding to the event week (February 13–19, 2023) were removed from the reference period.

Additionally, a temporal trend analysis was performed using the non-parametric theilSen method (Sen, 1968; Theil, 1950) to evaluate the concentration of PM before, during, and after the landslide event in São Sebastião, as well as to assess the statistical significance of the observed trends. The analysis was performed using the theilSen Function from the openair package (Carslaw, 2019; Carslaw & Ropkins, 2012). Three scenarios were defined for this analysis (Table 2), considering different n-days post-event periods (*n* = 1, 3, and 7 days), while the period of pre-event (*n* = 7 days) and event period (*n* = 2 days) remained constant.

**Table 2:** Description of the three analyzed scenarios in São Sebastião using the Theil-Sen method

Scenarios	Period			Total (*n*)
	**Pre-event (*n*)**	**Event (*n*)**	**Post-event (*n*)**	
1	11–17 Feb 2023 (7)	18–19 Feb 2023 (2)	20 Feb 2023 (1)	10
2	11–17 Feb 2023 (7)	18–19 Feb 2023 (2)	20–22 Feb 2023 (3)	12
3	11–17 Feb 2023 (7)	18–19 Feb 2023 (2)	20–26 Feb 2023 (7)	16

### Comparison with precipitation events

To assess whether the PM behavior observed during and after the February 2023 event was also observed during other high precipitation periods, two additional rainfall events were selected for comparison. June 13, 2023, was selected because it was identified as a prominent day within Cluster 3 and was positioned close to the February 18–19 event days in the cluster analysis, indicating similar meteorological characteristics and landslides as well. In contrast, March 19–20, 2022, was selected as a high precipitation event for which no landslides were identified in the available records, providing a comparative scenario without recorded mass movements. Daily PM_10_ and PM_2.5_ concentrations were compared with precipitation recorded at the four rain stations during and after the event.

## Results and discussion

### Analysis of meteorological conditions

**Fig. 3:**
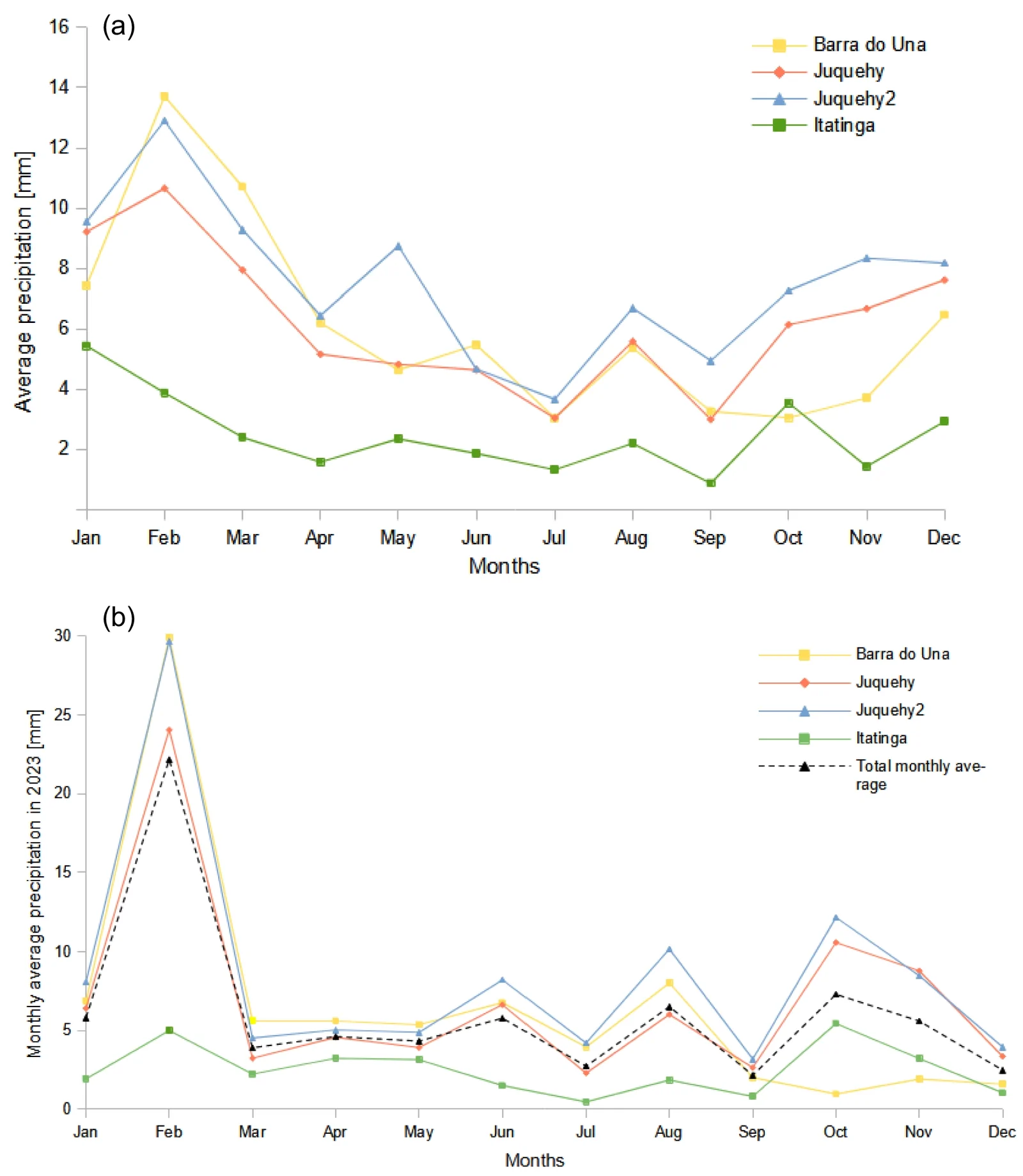
**a** Average monthly precipitation (mm) at the São Sebastião rain station from December 2021 to February 2025 and **b** Average monthly precipitation for each station and the overall average (mm) from January to December 2023

Precipitation in São Sebastião follows a clear seasonal pattern, typical of a humid tropical climate, such as that found along Brazil’s southeastern coast. The highest accumulated rainfall usually occurs between December and March, during the summer season, when atmospheric humidity and precipitation levels are higher (Fig. 3a). This pattern is associated with the influence of meteorological systems such as the South Atlantic Convergence Zone (SACZ) and cold fronts. These systems favor intense rainfall events (Rosa et al., 2020).

Of the events observed during the analyzed period, February 2023 stands out due to an extreme rainfall event that severely impacted the northern coast of São Paulo. This event was caused by the passage of an unusual cold front for the summer season that was intensified by the regional topography and the orographic effects of the Serra do Mar (INMET, 2023; Alcântara et al., 2025b). The accumulated precipitation exceeded the February 2023 average (Fig. 3b), contributing to landslide occurrences and severe socio-environmental impacts in several areas of São Sebastião. Additionally, the steep slopes of the Serra do Mar escarpments make the region even more susceptible to intense rainfall, fluvial processes, and both natural and human-induced mass movements (Alcântara et al., 2025b).

The intensity of this event was also influenced by the continuous inflow of humidity from the Atlantic Ocean toward the southeastern coast of Brazil. The large amount of water vapor available in the atmosphere favored the development of cloud formation and helped maintain precipitation over the region for an extended period. In addition, warmer-than-average ocean waters likely increased evaporation, providing even more humidity to the atmosphere. When these humid air masses reached the Serra do Mar, they were forced to rise due to the mountainous terrain, enhancing cloud formation and rainfall. Together, these factors contributed to the high precipitation totals recorded during the event and intensified its impacts on the region (Marengo et al., 2024).

Analysis of the rain stations revealed that the highest mean daily precipitation values were recorded in February at Barra do Una (29.92 mm) and at Juquehy2 (29.67 mm) (Fig. 3b). The Juquehy also recorded relatively high mean daily precipitation, with 24.04 mm in February 2023, while Itatinga recorded only 5 mm during the same month. These variations in precipitation reflect not only the influence of the seasonal cycle but also the effects of climate phenomena such as the transition from La Niña to neutral conditions in some Brazilian states including São Paulo, in February 2023 (INMET, 2023). Understanding these factors is crucial for predicting local climate behavior and for planning natural disaster prevention measures in vulnerable areas such as the São Paulo coast.

Beyond the specific meteorological drivers of the February 2023 event, climate change projections indicate that rainfall intensity in this region may continue to increase in the coming decades. Alcântara et al. (2025a) modeled landslide susceptibility in São Sebastião under four Intergovernmental Panel on Climate Change (IPCC) climate scenarios and found that the more severe scenarios correspond to a significant increase in areas with moderate to high landslide susceptibility, driven specifically by higher projected precipitation intensities. In line with this, Veloso et al. (2025) highlighted that municipalities along the Serra do Mar, including São Sebastião, have already experienced recurrent mass-movement disasters across consecutive rainy seasons (2022–2023 and 2023–2024), underscoring the growing urgency of early-warning and real-time monitoring systems in the region. Taken together, these findings suggest that the extreme rainfall event analyzed in this study may not be an isolated occurrence, but rather an early indication of a pattern that could become more frequent and more intense as climate change progresses with direct implications for landslide risk and, as the present study suggests, for the secondary air quality impacts associated with soil resuspension.

### Analysis of PM concentrations averages

**Fig. 4:**
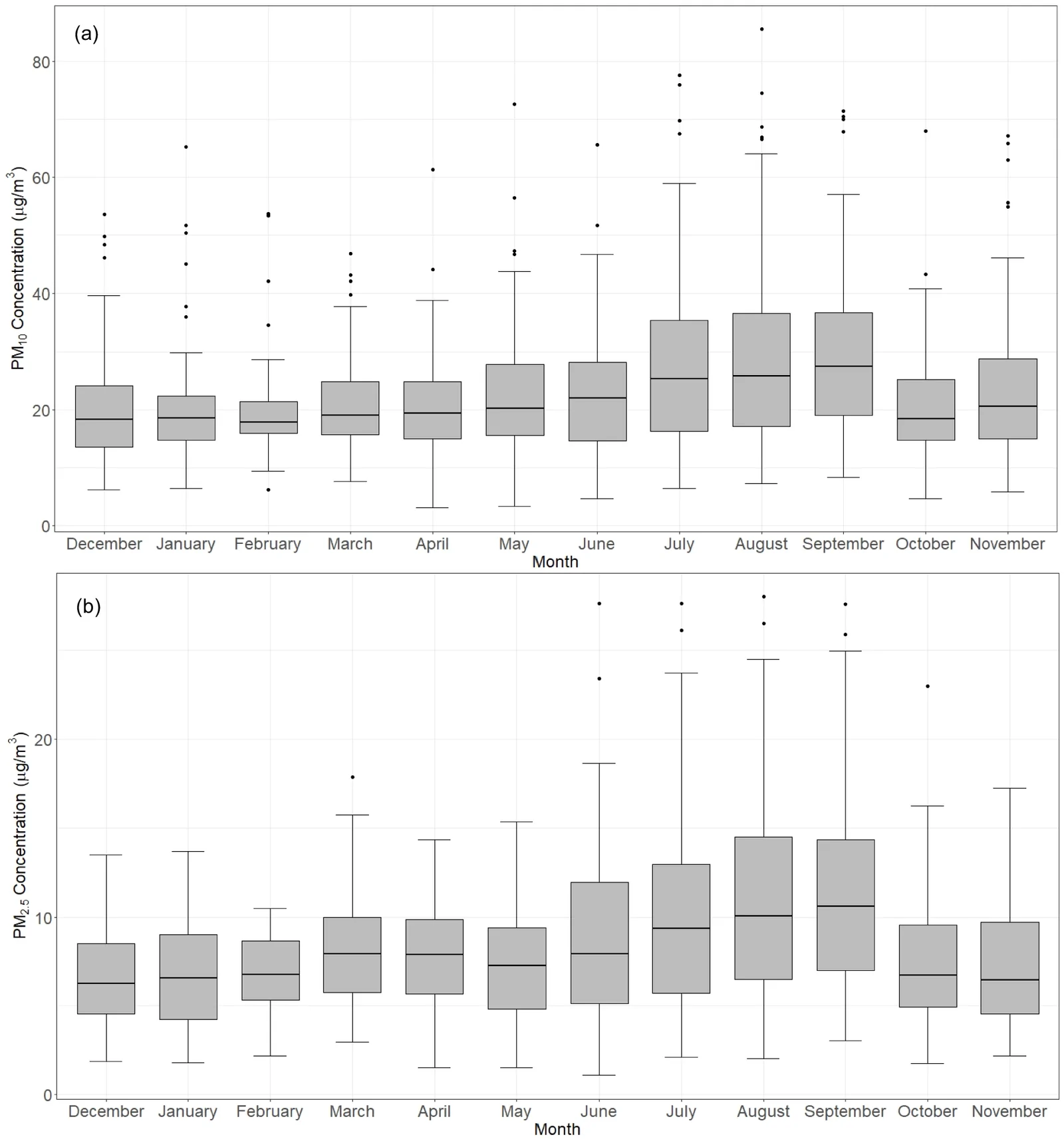
Boxplot of **a** PM10 daily concentration (µg/m^3^) and **b** PM2.5 daily concentration from December 2021 to February 2025

Figure 4a and b shows that between December 2021 and February 2025, the highest PM₁₀ and PM_2.5_ concentrations occurred mainly between July and September. This corresponds to the winter period and the early spring, when meteorological and local topographic conditions are less favorable for pollutant dispersion (Godoy & da Silva, 2022). A similar seasonal behavior has been reported in the Metropolitan Area of São Paulo (MASP), where Carvalho et al. (2015) observed higher monthly mean PM_10_ concentrations during winter. The authors also associate frequent episodes of elevated PM_10_ concentrations in the MASP mainly with vehicular emissions, including primary particle emissions and processes involved in the formation of secondary particles. Similar seasonal patterns have also been reported in studies conducted in South Korea (Lee et al., 2023) and China (Li et al., 2018). These studies showed that the average PM concentration and frequency of episodes tend to be higher in winter than in other seasons.

In the case of São Sebastião, the presence of the Serra do Mar may significantly impact the local atmospheric circulation. During the winter, a combination of factors, including complex terrain and weaker lower-level wind circulation, contributes to the accumulation of pollutants in urban areas, resulting in higher pollutant levels. Regions with similar topographic and atmospheric conditions can show comparable PM_2.5_ pollution patterns. Zhu et al. (2023) identified secondary aerosol formation, industrial emissions, and vehicular emissions as important sources of PM_2.5_ in the studied region. In areas surrounded by mountains, limited air circulation can also favor the accumulation of locally emitted pollutants and the transport of PM from nearby areas.

Farias (2013) observed that, during summer, greater atmospheric instability in the Metropolitan Region of Rio de Janeiro (MRRJ) favors the upward movement of pollutants. These pollutants are then transported inland by sea breeze circulation. The Serra do Mar acts as a barrier, retaining the pollutants within air basins. During the winter, atmospheric stability combined with a shallower mixing layer keeps pollutants closer to the surface. This concentrates them over the MRRJ due to the influence of the South Atlantic Subtropical Anticyclone (SASA). A similar influence of coastal circulation and complex topography has been observed in the Greater Vitória Region, Brazil, where local relief and sea-breeze dynamics affect atmospheric circulation and, consequently, pollutant transport and dispersion. Under sea-breeze conditions, the formation of a thermal internal boundary layer (TIBL) can further limit vertical mixing and favor pollutant accumulation near the surface (Salvador et al., 2016).

**Fig. 5:**
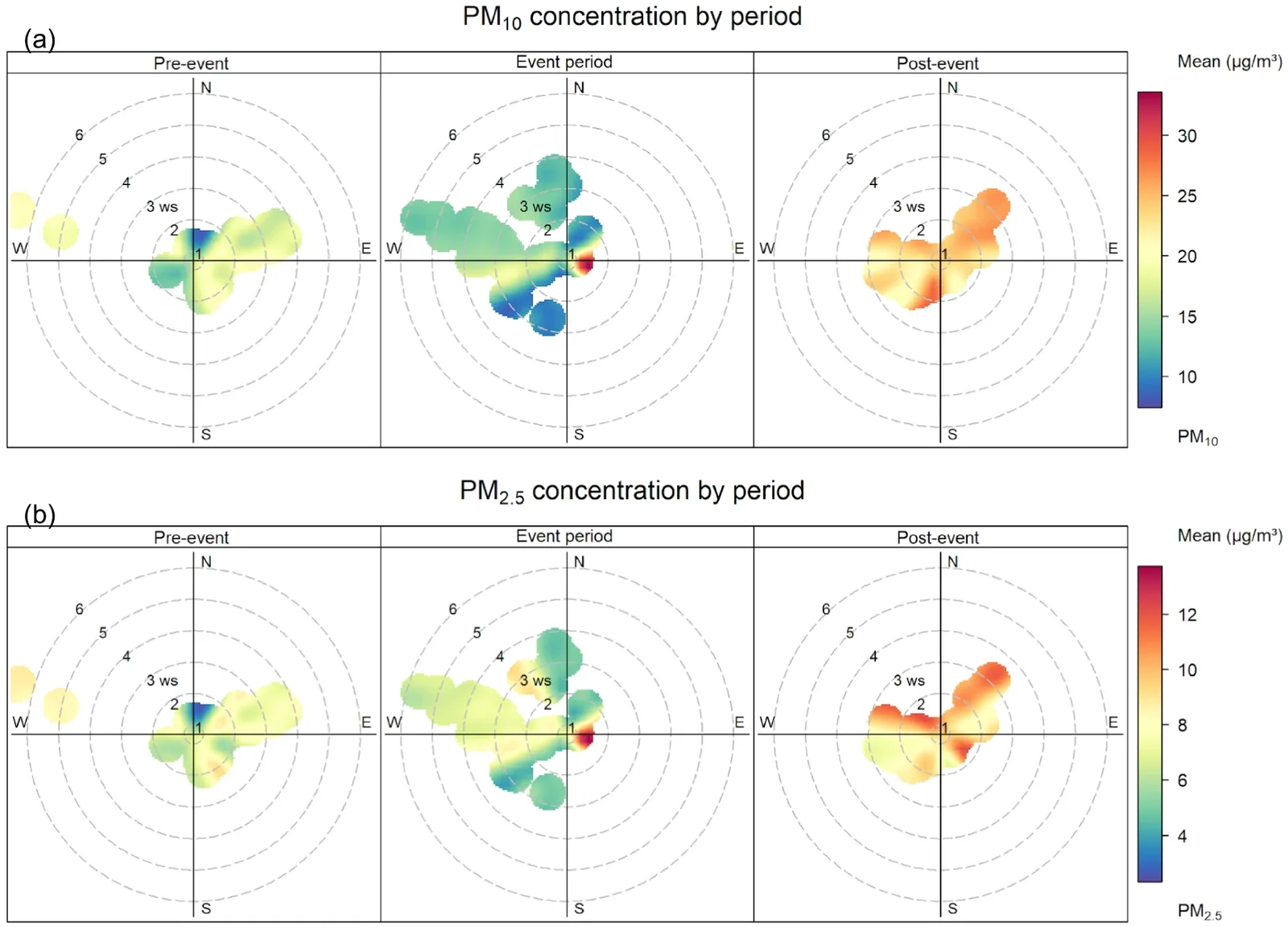
Polar plots of the mean **a** PM10 and **b** PM2.5 concentrations (µg/m^3^) as a function of wind direction and wind speed. The data are divided into three periods: pre-event, event, and post-event. Each period lasts 2 days in São Sebastião

Figure 5 shows the polar plots of the mean concentrations of PM_10_ and PM_2.5_ according to wind direction and speed. The plots are divided into three periods: 2-day lag before the event, event, and 2-day lag after the event. Each covering 2 days. A clear transition was observed: relatively low and dispersed concentrations during the pre-event period to a significant accumulation during the event, and persistence of high concentrations in the post-event period.

In the pre-event period, PM_10_ and PM_2.5_ concentrations were generally lower than those observed after the event and were mainly associated with low to moderate wind speeds, mostly below 3 m/s. The concentration pattern showed contributions from both eastern and western wind directions, suggesting the influence of more than one transport pathway before the extreme event.

During the event period, both PM_10_ and PM_2.5_ exhibited pronounced peaks coinciding with low wind speeds (below 1 m/s) predominantly from the east-southeast. This is likely related to the timing of the rainfall: since precipitation only began on the evening of February 18, concentrations remained elevated throughout the morning hours, when no rain had yet occurred to promote pollutant removal. This pattern strongly suggests atmospheric stagnation, in which reduced ventilation limits the dispersion of pollutants and leads to their local accumulation (Lee et al., 2023). However, the analysis revealed a different pattern in the northwestern (NW) region of the municipality, where wind speeds above 6 m/s were consistently associated with low concentrations of both pollutants. This pattern supports the hypothesis that the Serra do Mar, located west of the municipality, may reflect the influences of local atmospheric circulation through orographic effects. However, because the polarPlot function summarizes concentrations according to wind speed and direction, it does not explicitly show how concentrations evolved throughout the event.

**Fig. 6:**
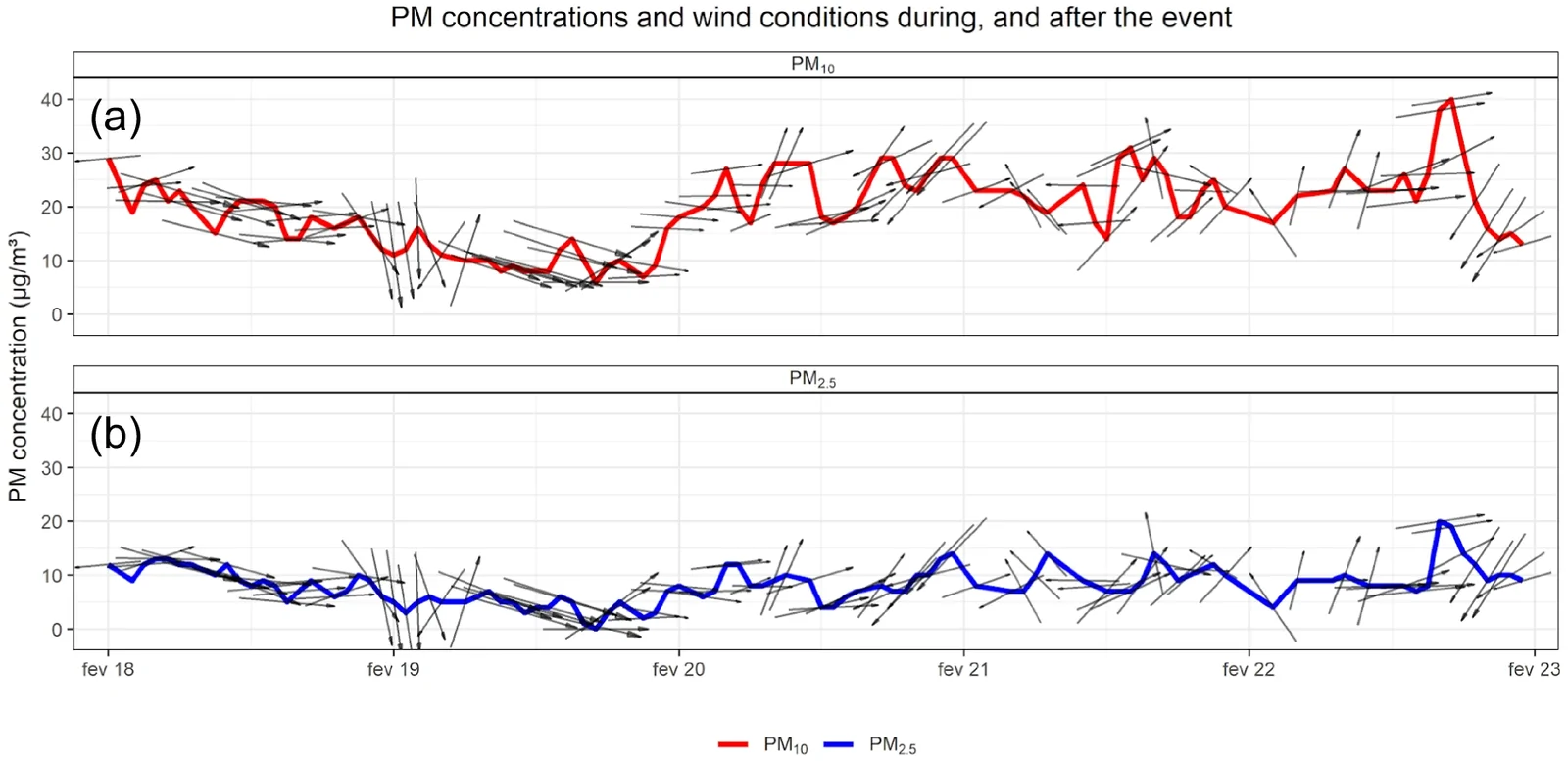
Timeplot with hourly data of **a** PM_10_ and **b** PM_2.5_ concentrations (µg/m^3^) as a function of wind direction and wind speed. The data cover the event and post-event periods from February 18 to 22, 2023

To provide additional temporal information, Fig. 6 shows the hourly evolution of PM_10_ and PM_2.5_ together with wind vectors during, and after the event. A progressive decrease in both PM fractions was observed during the event, with the lowest concentrations occurring toward the end of the event. This behavior suggests that the high concentrations depicted in Fig. 5 were associated with wind conditions during specific hours before the event, rather than with sustained PM accumulation throughout the entire event period.

The decrease in PM concentration coincided with the development of intense rainfall and changes in wind conditions. Precipitation can promote PM removal through wet scavenging, although the magnitude of this effect depends on particle size, rainfall characteristics, initial PM concentrations, and meteorological conditions (Liu et al., 2020).

A contrasting pattern emerged after the event. PM concentrations increased rapidly and remained elevated during the following days, with particularly high hourly concentrations observed on February 22. The polarPlot analysis (Fig. 5) showed that these higher post-event concentrations were mainly associated with low to moderate wind speeds, conditions that are generally less favorable for atmospheric dispersion. However, elevated concentrations occurred under several wind directions rather than being restricted to a single sector. Liu et al. (2020) showed that the influence of wind on PM_10_ and PM_2.5_ depends on the interaction between wind speed and direction. While weaker winds may limit atmospheric dispersion and favor local pollutant accumulation, winds from specific sectors may also transport PM from upwind sources. Therefore, wind speed alone is unlikely to explain the post-event increase observed in São Sebastião.

The extreme rainfall and landslide event occurred during the 2023 Carnival holiday period, when a substantial increase in road traffic was expected along the northern coast of São Paulo. According to estimates from DER-SP (2023), approximately 223,718 vehicles were expected to travel along the SP-055 Highway during the Carnival period. In addition, between February 21 and 22, eastbound daily traffic volumes approximately doubled (approximately 6,395 each day) compared with typical values observed during the rest of the month. In this context, the increased post-event concentrations may be partly associated with PM resuspension. However, this hypothesis cannot be confirmed based on the available data, as other factors associated with the landslide event may also have contributed, including increased traffic related to rescue operations, the circulation of heavy machines, excavators, trucks, and vehicles involved to remove mud and debris and restoring access to the affected area. Moreover, emissions from nearby activities, including operations at the Port of São Sebastião, as well as associated vessel and road traffic, may also have contributed to local PM concentrations.

**Fig. 7:**
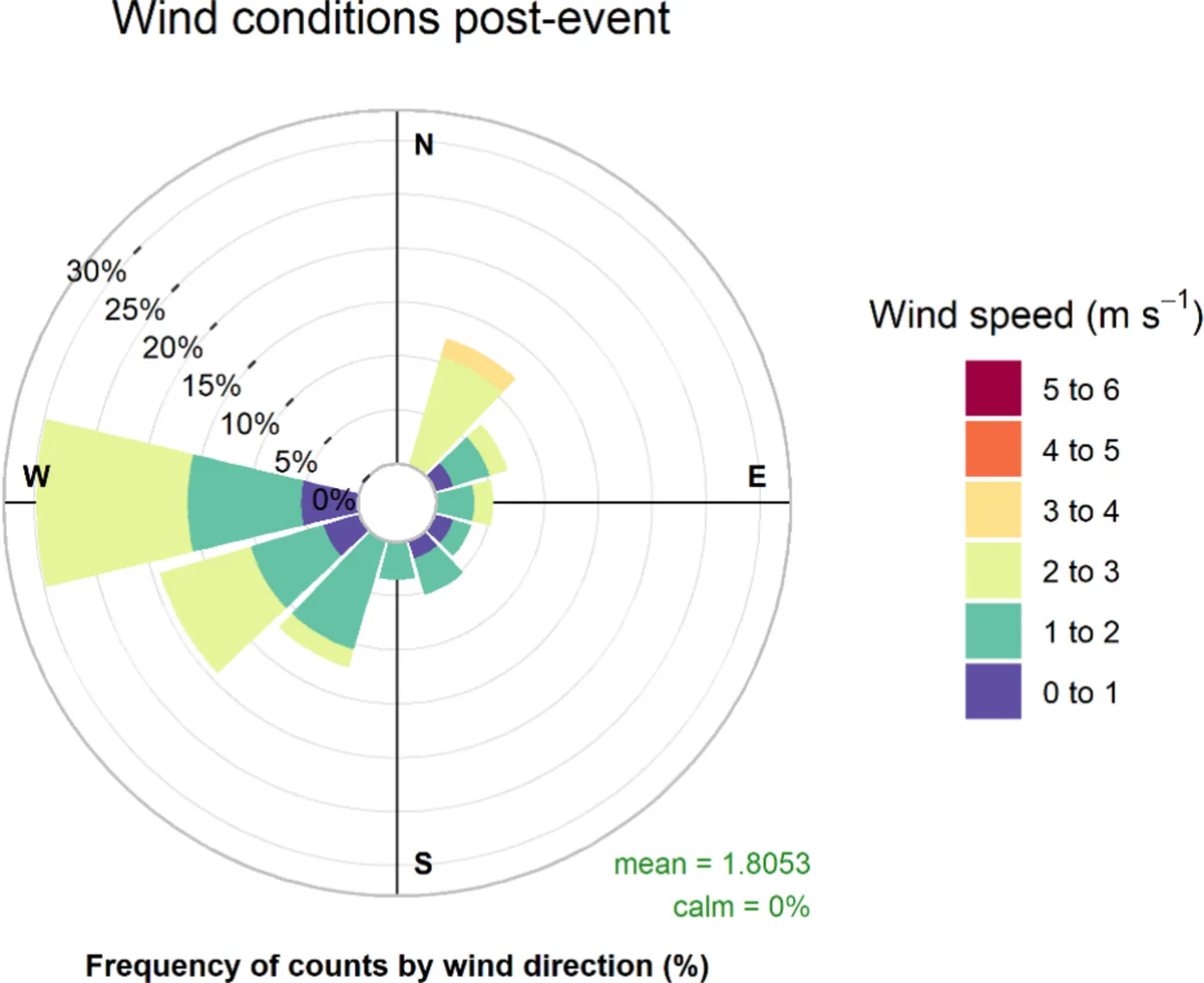
windRose function showing the frequency distribution of wind direction and wind speed. The analysis was performed for the post-event period (February 20–22, 2023)

To better characterize wind conditions during the days following the event, an additional analysis was performed for the post-event period (February 20–22, 2023), using hourly wind speed and wind direction data. Figure 7 presents the results of the windRose analysis. Westerly winds were the most frequent, accounting for approximately 30% of the observations and occurring predominantly at wind speeds between 1 and 3 m/s. Winds from the southwestern region were also frequent, indicating a clear predominance of winds from the western and southwestern areas during the post-event period. Overall, wind speeds were generally low to moderate, with a mean value of approximately 1.8 m/s.

Overall, the polarPlot, timePlot, and windRose analyses indicate that the post-event increase in PM cannot be attributed to a single wind direction or transport pathway. Weaker winds may have contributed to reduced atmospheric dispersion, while the occurrence of elevated concentrations under different directional sectors suggests the simultaneous influence of multiple local and regional sources. The recurrent presence of westerly and southwesterly winds is compatible with transport from areas located west of the monitoring station, including the landslide-affected region. However, the available data do not allow a definitive attribution of the measured PM to this source.

### Statistical analysis

For the CA application, only days with complete measurements of all analyzed parameters were considered. However, PM was excluded at this stage to ensure statistical consistency and comparability between the datasets. Consequently, 28.41% of the data, or 337 days out of 1187, were excluded from the initial stage of the study. This underscores the importance of maintaining properly functioning monitoring stations, as continuous and reliable data are crucial for evaluating environmental impacts, identifying extreme events, and supporting effective risk management.

The *k*-means method was selected to identify meteorological patterns during the study period. The clustering procedure considered the daily frequency of the data throughout each year, and values of *k* ranging from two to five were tested. A Silhouette analysis indicated *k* = 2 as the solution with the highest overall cohesion and separation, with an average Silhouette width of approximately 0.50, compared with approximately 0.24 for *k* = 3. Using the Elbow method, the optimal value chosen was *k* = 3. This value was selected when the relative reduction in intra-cluster variance dropped below 10%. This reduction is calculated as $\frac{\left({WSS}_{k}-{WSS}_{k+1}\right)}{{WSS}_{k}}$, where *WSS*_*k*_ is the total intra-cluster sum of squares for *k* clusters. Thus, this solution (*k* = 3) provided a finer partition of the data, isolating a smaller group characterized by high rainfall that included both event days.

**Fig. 8:**
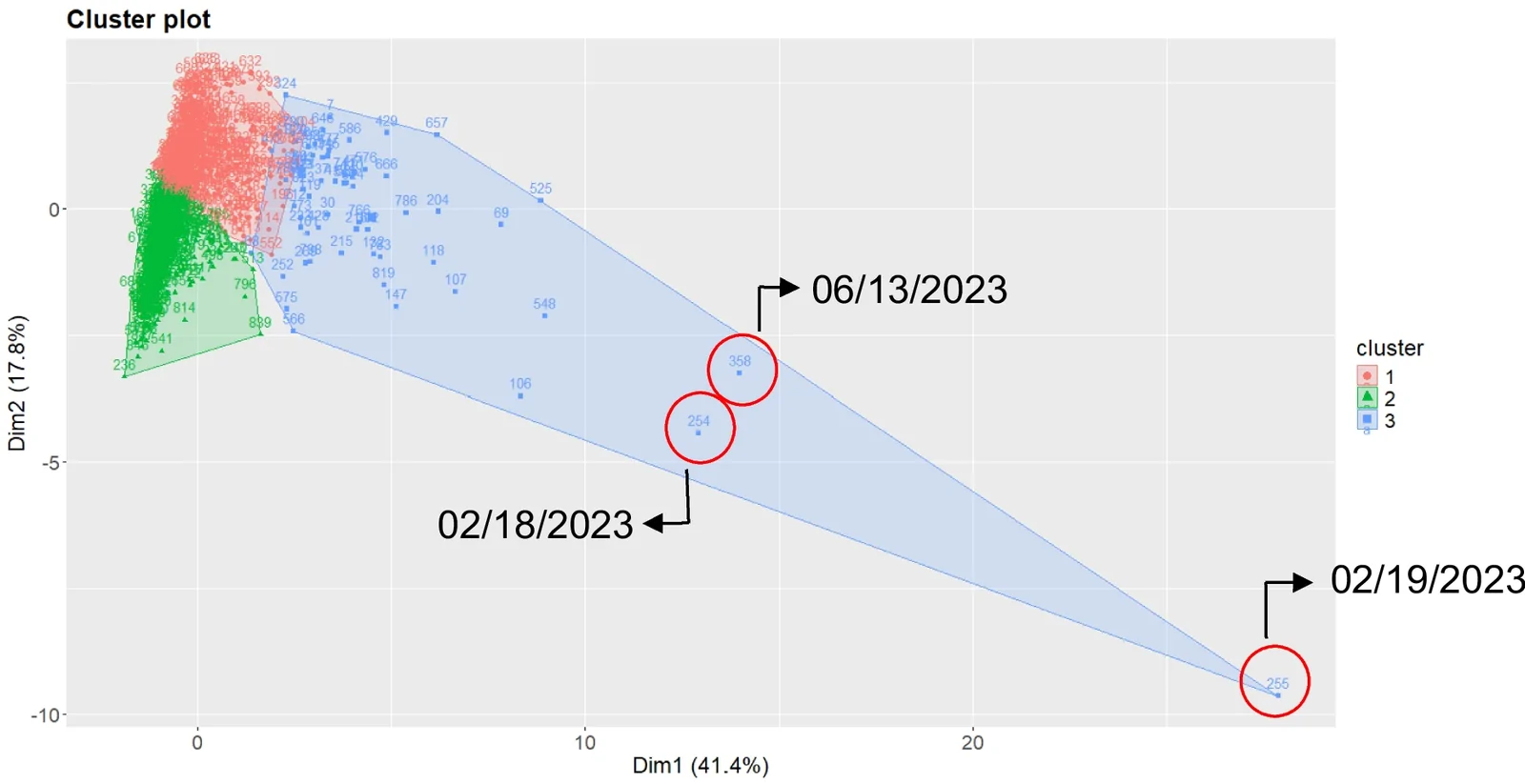
Clusters identified by the k-means method. Clusters 1 and 2 represent more common meteorological conditions, while Cluster 3 (the control group) includes days with more significant meteorological variability and extreme precipitation events

Cluster 3 was defined as the “control group” and chosen for further analysis because it included the days on which landslides occurred, as well as other days with meteorological conditions. Of the days classified in the control group, three records (254, 255, and 358) were significantly different from the other data within the same group, as shown in Fig. 8. Record 255 corresponds to the event day, February 19, 2023, and had the highest maximum precipitation, at 450.03 mm, compared to the others. Record 254 corresponds to the one day before the event (February 18, 2023), which had a maximum precipitation of 198.70 mm. Record 358 corresponds to June 13, 2023, which had a maximum precipitation of 214.26 mm. These results suggest that, during the analyzed period, event day exhibited a unique precipitation pattern compared to the other days during the analyzed period. Additionally, the highest mean precipitation in the control group was recorded at the Juquehy2 rain station (Table 3), with 158.94 mm.

**Table 3:** Minimum, mean, and maximum values for each cluster and parameter. This table uses daily data obtained using the *k*-means method

**Variable**		**Cluster**	**Minimum**	**Mean**	**Maximum**
**Temperature [ºC]**		1	17.83	25.36	33.10
		2	14.80	22.83	31.18
		3 (control group)	20.97	23.90	27.43
**Wind direction [º]**		1	49.00	135.14	305.67
		2	64.91	219.87	336.33
		3 (control group)	137.67	231.89	291.75
**Relative humidity [%]**		1	42.13	76.12	98.54
		2	65.25	87.22	99.00
		3 (control group)	80.88	88.60	97.58
**Wind speed [m/s]**		1	1.03	2.29	4.36
		2	0.98	1.98	4.27
		3 (control group)	1.40	2.24	2.99
**Precipitation [mm]**	**Barra do Una**	1	0.00	1.69	42.,30
		2	0.00	12.40	77.79
		3 (control group)	20.85	149.08	450.03
	**Juquehy**	1	0.00	1.25	32.39
		2	0.00	12.27	79.69
		3 (control group)	70.45	134.95	339.93
	**Juquehy2**	1	0.00	1.49	39.40
		2	0.00	14.46	101.86
		3 (control group)	88.73	158.94	414.36
	**Itatinga**	1	0.00	0.60	14.62
		2	0.00	5.76	73.52
		3 (control group)	0.00	19.48	52.83

Regarding the meteorological variations (Table 3), Cluster 1 exhibited the highest mean temperatures (25.36°C) and elevated maximum temperatures (33.10°C). These temperatures were associated with the lowest mean relative humidity values, indicating warmer and potentially more unstable conditions. Cluster 2 had the lowest mean temperatures (22.83°C) and intermediate relative humidity values, while the control group showed intermediate mean temperatures (23.90°C) and higher relative humidity values. Wind direction varied considerably between the clusters, while wind speed remained relatively low in all groups, with mean values below 2.3 m/s. This condition may favor lower pollutant dispersion.

A high contrast was observed in the precipitation between the clusters. Cluster 1 was characterized by minimum values of zero and very low mean precipitation values at all rain stations, representing predominantly dry periods. Cluster 2 showed intermediate mean precipitation values and frequent days without rainfall. In contrast, the control group had the highest mean and maximum precipitation values across all stations, with expressive maximum values indicating the occurrence of intense rainfall events.

The data within the control group were divided into two categories: the “Days of the Event” (DE) category, which corresponded to February 18 and 19, 2023 (days associated with the extreme rainfall event in São Sebastião), and the “Remaining Days” (RD) category, which comprised the other days classified in the control group that exhibited similar meteorological patterns.

**Fig. 9:**
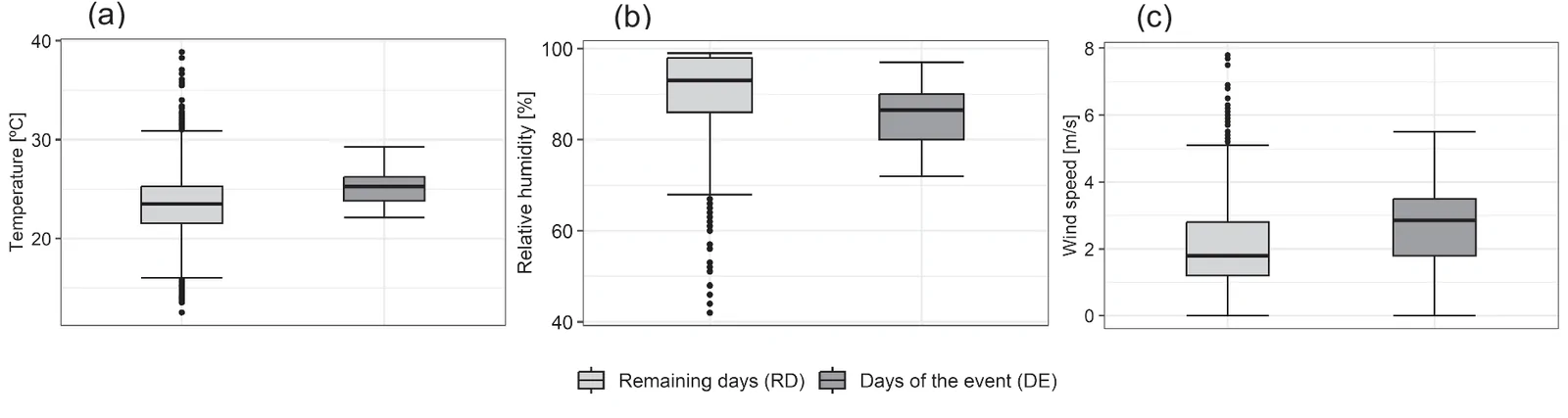
Boxplot of hourly precipitation meteorological variables: **a** temperature, **b** relative humidity, and **c** wind speed. This figure uses hourly data from the control group obtained with the k-means method

Based on this division, the analysis considered four meteorological variables: temperature, relative humidity, precipitation, wind speed, and wind direction. Figure 9a and c demonstrates that during the DE event, the temperature and wind speed were slightly higher. These results suggest that although the differences were not very extreme, the event days showed greater thermal stability and lower data dispersion around the median. However, Zender-Świercz et al. (2024) observed a weak correlation between temperature and PM concentrations, indicating that temperature alone does not strongly influence PM levels and the correlation can vary by location and air quality conditions.

According to Zender-Świercz et al. (2024), relative humidity (Fig. 9b) may contribute to PM accumulation through hygroscopic growth and secondary aerosol formation. The authors reported positive Spearman correlations between relative humidity and PM concentrations, with the strength of the relationship varying according to air quality conditions. Under good air quality levels (0–20 μg/m^3^ for PM_10_ and 0–13 μg/m^3^ for PM_2.5_), weak positive correlations were observed for both PM_10_ and PM_2.5_ (*r*_*s*_ = 0.2), whereas under fair and moderate air quality levels (20.1–50 μg/m^3^ for PM_10_ and 13.1–35 μg/m^3^ for PM_2.5_), strong positive correlations were found for PM_10_ (*r*_*s*_ = 0.7) and PM_2.5_ (*r*_*s*_ = 0.8). No correlation was observed under extremely poor air quality conditions (*r*_*s*_ = 0.0). In the present study, relative humidity was lower during DE, while median PM concentrations were slightly higher. This suggests that relative humidity alone does not explain the observed behavior during the event. Although relative humidity was not particularly elevated when compared to the RD, the event occurred under conditions that favored the continuous supply of humidity to the region (Marengo et al., 2024).

**Fig. 10:**
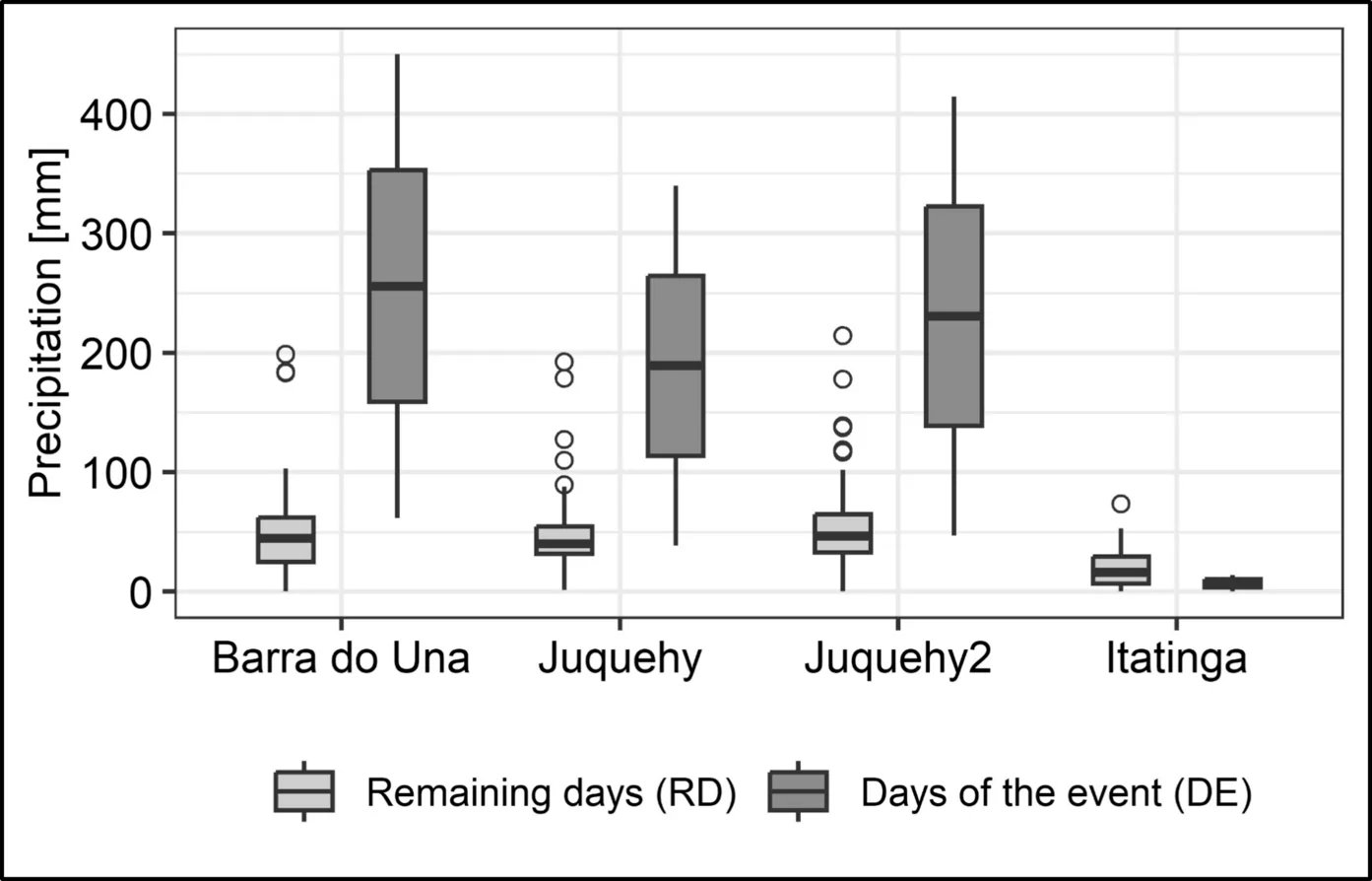
Boxplot of the daily precipitation data from the Barra do Una, Juquehy, Juquehy2, and Itatinga rain stations for the control group obtained using the *k*-means method

Figure 10 compares precipitation data from four rain stations (Barra do Una, Itatinga, Juquehy, and Juquehy2). At the three stations closest to the event area, the median precipitation value was close to zero during RD, indicating that most of these days were dry or had insignificant rainfall. In contrast, the DE showed much higher median precipitation values.

Therefore, the days associated with the event were characterized by a combination of meteorological conditions, including lower relative humidity, stronger winds, and intense precipitation. Together, these conditions define a period of intense rainfall impact when compared to RD. Combined with the influence of the Serra do Mar and the persistence of rainfall over the same area, these factors contributed to the high precipitation totals observed during the event (Marengo et al., 2024).

**Fig. 11:**
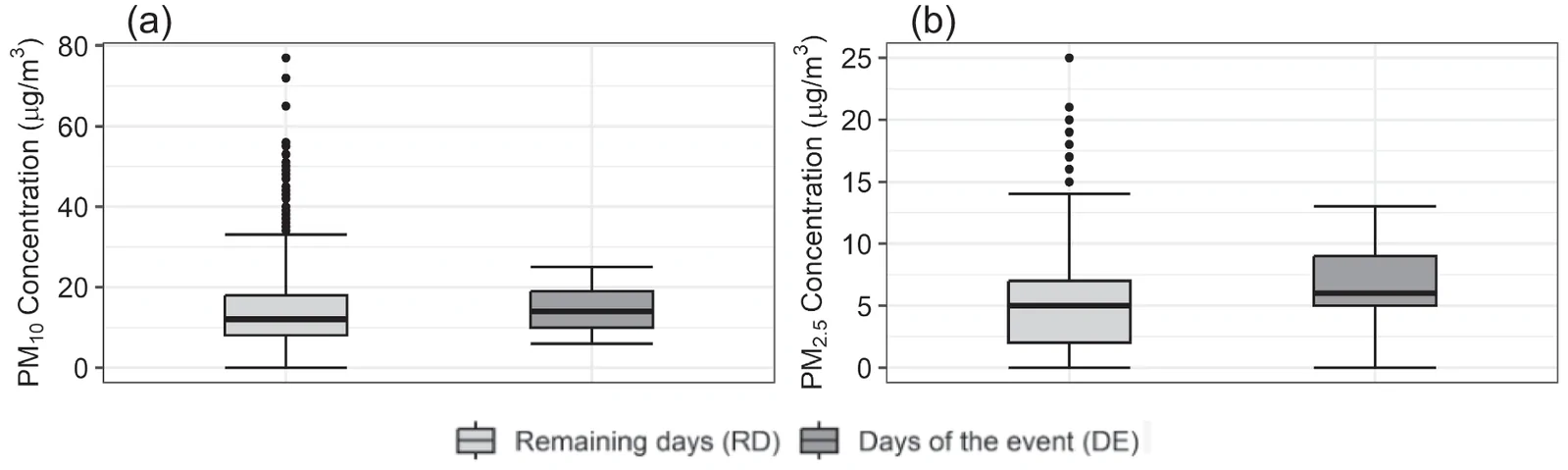
Boxplot of hourly **a** PM_10_ and **b** PM_2.5_ data for the control group obtained using the *k*-means method

Figure 11 shows that both PM_10_ and PM_2.5_ concentrations were higher during DE for the data classified in the control group. It is important to note that PM was not used as an input variable in the cluster analysis. Therefore, the clusters were defined based solely on meteorological variables. The PM concentrations on those days were evaluated afterwards. This discrepancy with the anticipated washout effect brought on by heavy rainfall that began on the night of February 18, can be attributed to the use of daily mean values, which fail to capture short-term reductions in pollutant concentrations. A subsequent analysis will further explore PM behavior before, during, and after the event to provide a more detailed interpretation.

### Temporal analysis of PM

**Fig. 12:**
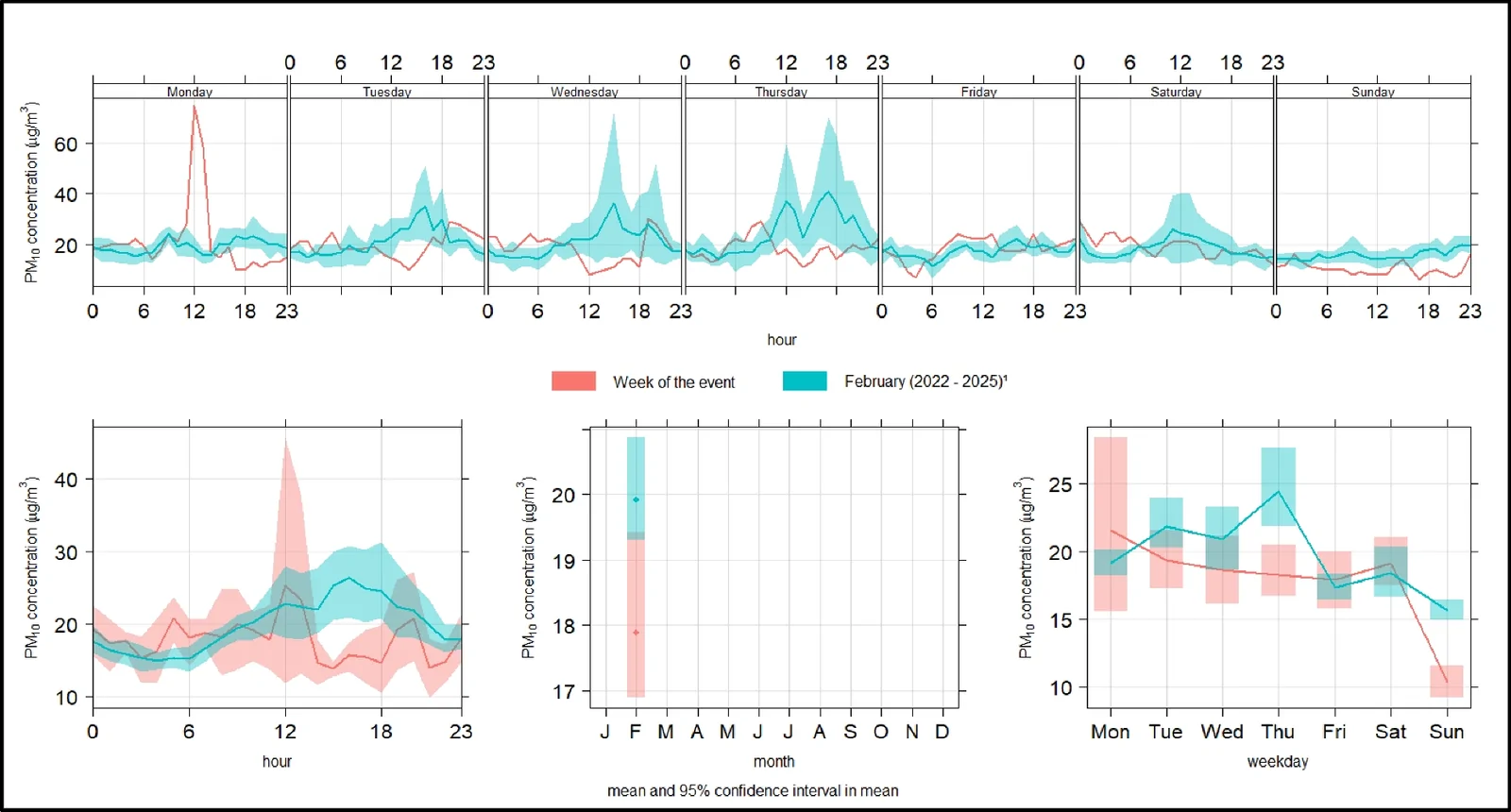
PM_10_ concentrations during the event week and throughout February between 2022 and 2025. ^1^The reference period includes PM_10_ concentration data from every February between 2022 and 2025, except for the measurements taken during the week of the event (February 13–19, 2023)

**Fig. 13:**
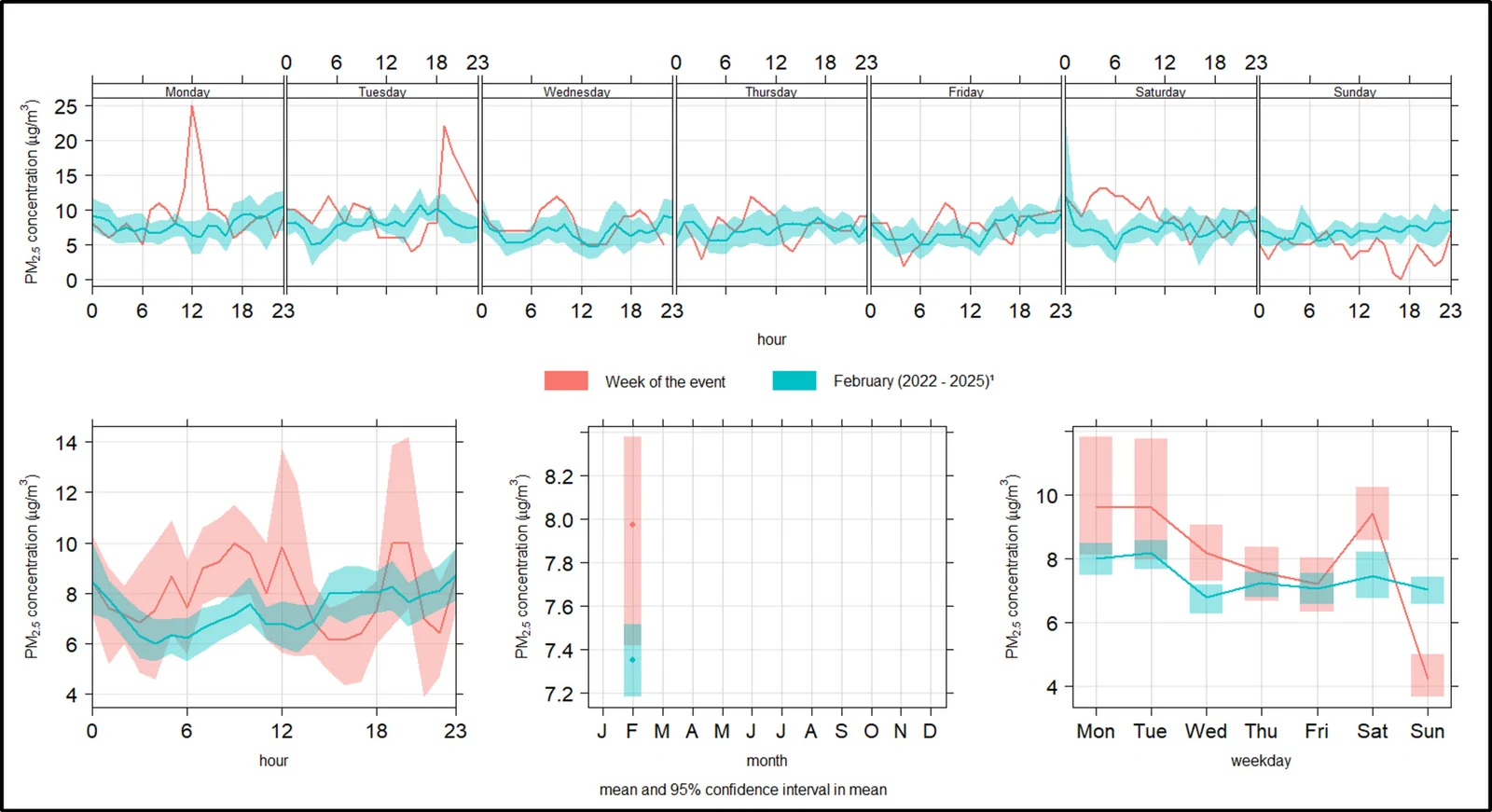
PM_2.5_ concentrations during the event week and throughout February between 2022 and 2025

Figures 12 and 13 show a comparison of PM_10_ and PM_2.5_ concentrations during the event week, between February 13 and 19, 2023 (Monday–Sunday), and during a reference period. The reference period consisted of PM data from every February between 2022 and 2025, excluding the data from the event week.

The results show that PM_10_ and PM_2.5_ levels decrease significantly during days with intense rainfall, especially on Sunday afternoon, February 19, 2023. This is mainly because precipitation acts as a wet removal mechanism, whereby rainfall washes the atmosphere and removes suspended particles. This prevents the high pollution levels normally associated with human activities (Wang et al., 2023), as evidenced by the average February values between 2022 and 2025. Additionally, Fig. 9 shows that the DE had stronger winds than the RD. Stronger winds help spread PM in the air (Lee et al., 2023) by mixing the air more thoroughly and moving pollutants horizontally, which reduces their concentration near the surface.

As for QUALAR air quality patterns, PM concentrations remained below the NAQS throughout the event week (PM_10_ = 120 µg/m^3^ and PM_2.5_ = 60 µg/m^3^ over a 24-h period). This behavior is consistent with the impact of the heavy rains at the end of the week and the time of the year. Concentrations are naturally lower during this period (PM_10_ ≈ 20 μg/m^3^ and PM_2.5_ ≈ 8 μg/m^3^). This differs from the higher levels observed in winter, when stable atmospheric conditions hinder the dispersion of pollutants and facilitate their accumulation (Godoy & da Silva, 2022).

**Fig. 14:**
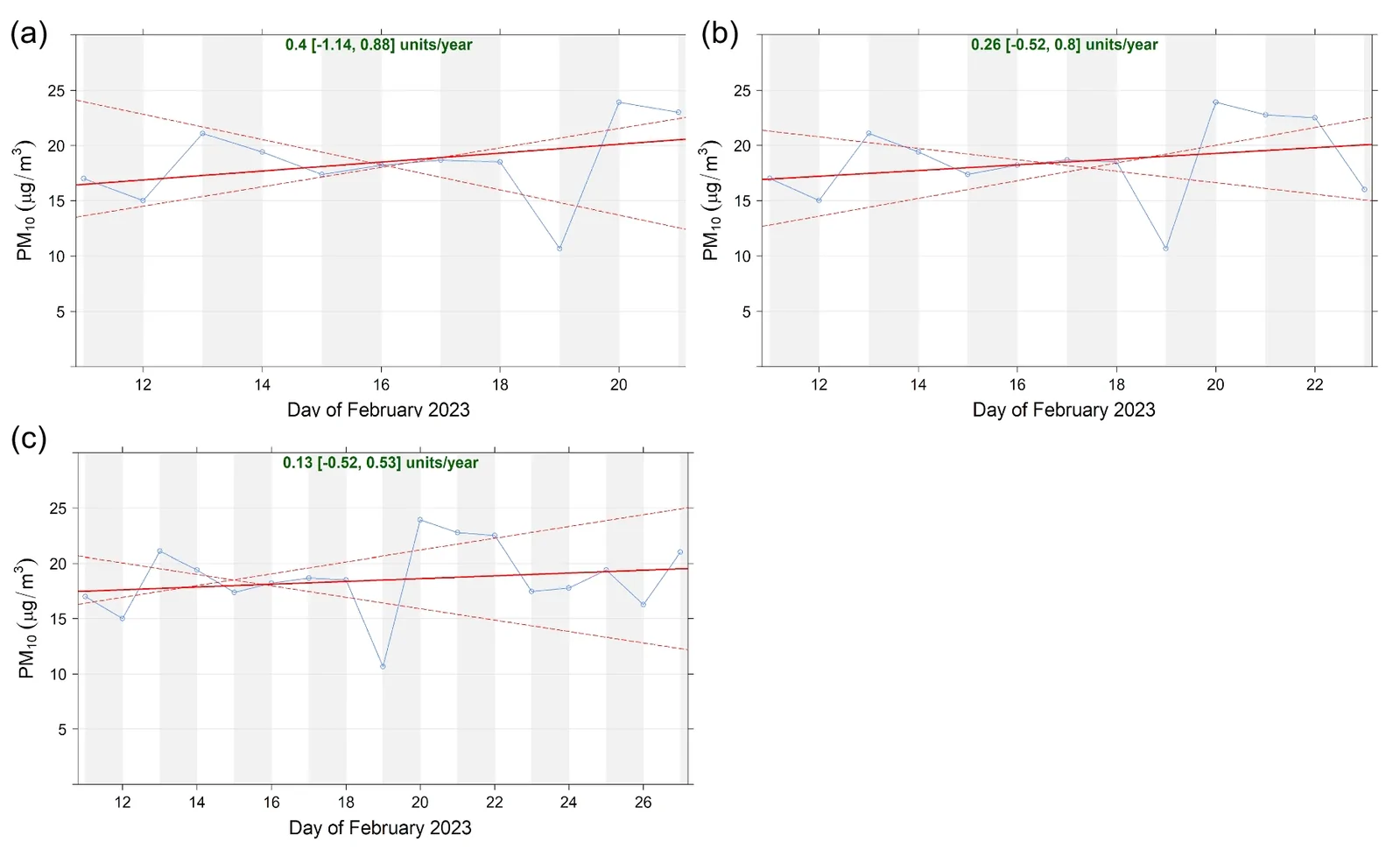
Temporal trend of daily PM_10_ concentrations for **a** February 11–20, 2023, **b** February 11–22, 2023, and **c** February 11–26, 2023

**Fig. 15:**
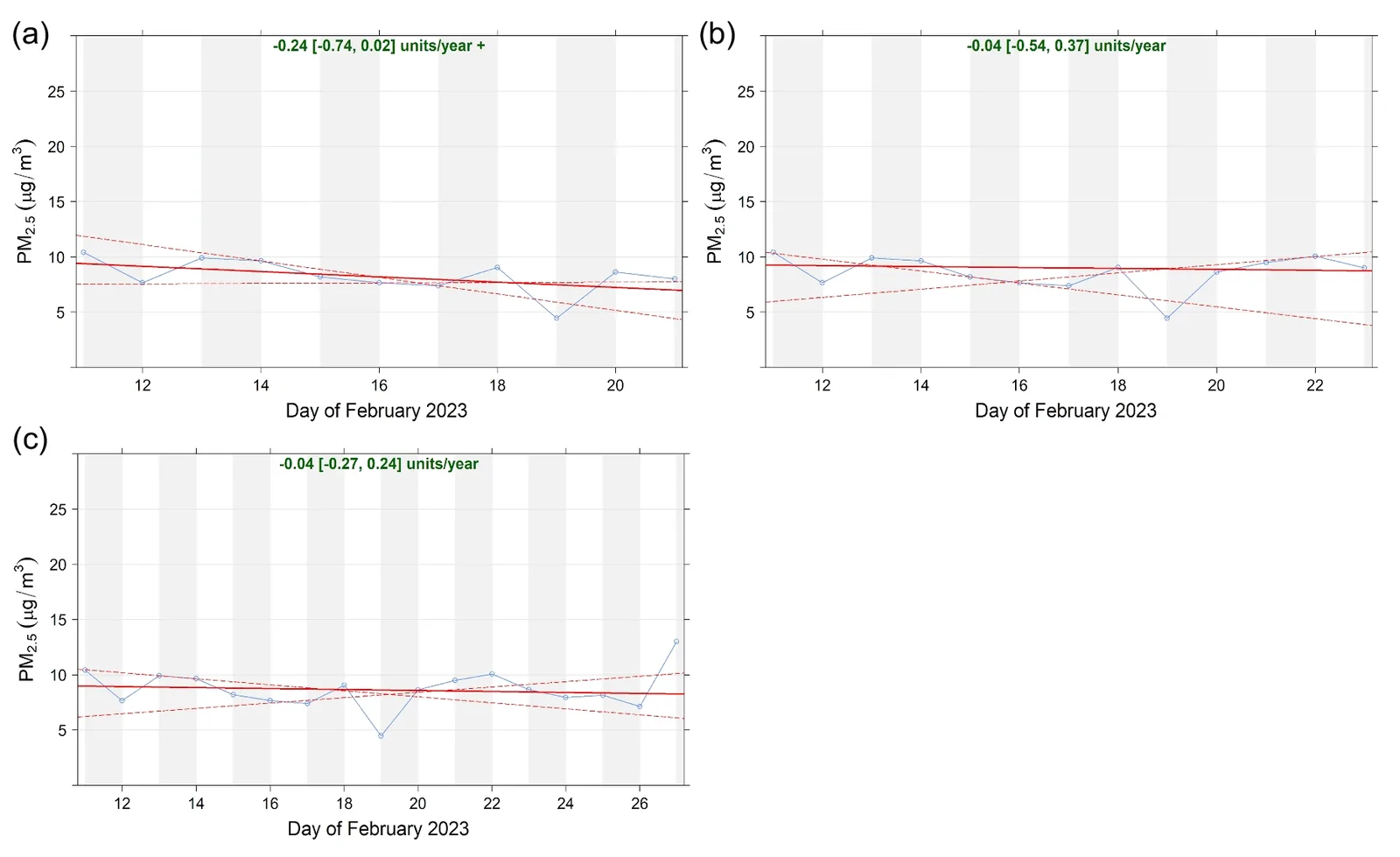
Temporal trend of daily PM_2.5_ concentrations for **a** February 11–20, 2023, **b** February 11–22, 2023, and **c** February 11–26, 2023

In addition, the Theil-Sen analysis (Figs. 14 and 15) was used to evaluate the temporal trends of daily PM_10_ and PM_2.5_ concentrations, respectively, during the landslide period, which included the 7 days before and up to 7 days after the event (Table 2). Different temporal scenarios were considered (1, 3, and 7 days after the event) to investigate possible changes in trend slopes and estimate the time required for concentrations to return to their normal levels or increase again.

In all scenarios studied, the calculated slopes were close to zero, and the confidence intervals included the null value. This indicated that the short-term trend was statistically nonsignificant. This suggests that short-term variations predominated, without consistent increases or decreases in daily PM concentrations.

Rainfall began around 7:00 p.m. on February 18, indicating a transition to more unstable atmospheric conditions. During the first three hours (7:00 p.m. to 10 p.m.) after the rainfall began, there was a slight increase in particulate matter concentrations, with PM_10_ ranging from 16 to 18 μg/m^3^ and PM_2.5_ ranging from 6 to 10 μg/m^3^. The most critical period of the event occurred between 9:00 p.m. on February 18 and 4:00 a.m. on February 19, with high hourly accumulated precipitation recorded at all stations, ranging from 34.31 mm (Juquehy at 9:00 p.m.) to 101.59 mm (Barra do Una at 12:00 a.m.). During this time, consistent reductions in PM_10_ (6–16 μg/m^3^) and PM_2.5_ (0–7 μg/m^3^) concentrations were observed, which highlights the role of wet deposition in removing atmospheric particles. After 11:00 p.m. on February 19, when rainfall weakened or stopped at most stations and remained only as light precipitation at Juquehy2 (with a maximum of 0.4 mm), PM concentrations increased again. This suggests reduced atmospheric washout efficiency and the resumption of pollutant accumulation. From February 19 to February 20, PM_10_ concentrations increased by 124% (from 10.67 to 23.91 μg/m^3^), and PM_2.5_ concentrations by 93.3% (from 4.46 to 8.62 μg/m^3^).

McMullen et al. (2021) observed this behavior. After rainfall events, concentrations of fine particles (0.2–3 μm) may increase and remain elevated for up to 20 h. These increases range from 13 to 35% and are associated with the resuspension and atmospheric redistribution of deposited material. Concentration of intermediate particles (3–10 μm) tends to increase during the first hours and then returns to previous levels within approximately six hours. In contrast, larger particles (10–20 μm) initially tend to decrease due to wet deposition and then gradually increase due to surface resuspension. These granulometric differences indicate that concentration recovery after precipitation may occur over timescales ranging from hours to several tens of hours, which explains the rapid fluctuations observed in the estimated PM_10_ and PM_2.5_ temporal curves in this study.

Zheng et al. (2019) reported similar results regarding the influence of precipitation characteristics. They demonstrated that light, moderate, and intense rainfall events have a positive removal effect on PM_10_ and PM_2.5_, with increased efficiency associated with higher intensity and duration of rainfall. In contrast, drizzle events may have a negative removal effect, temporarily increasing concentrations. The authors also reported that longer rainfall events promote greater removal efficiency and extend the period of reduced concentrations. They noted seasonal differences and the greater efficiency of nighttime precipitation as well.

**Fig. 16:**
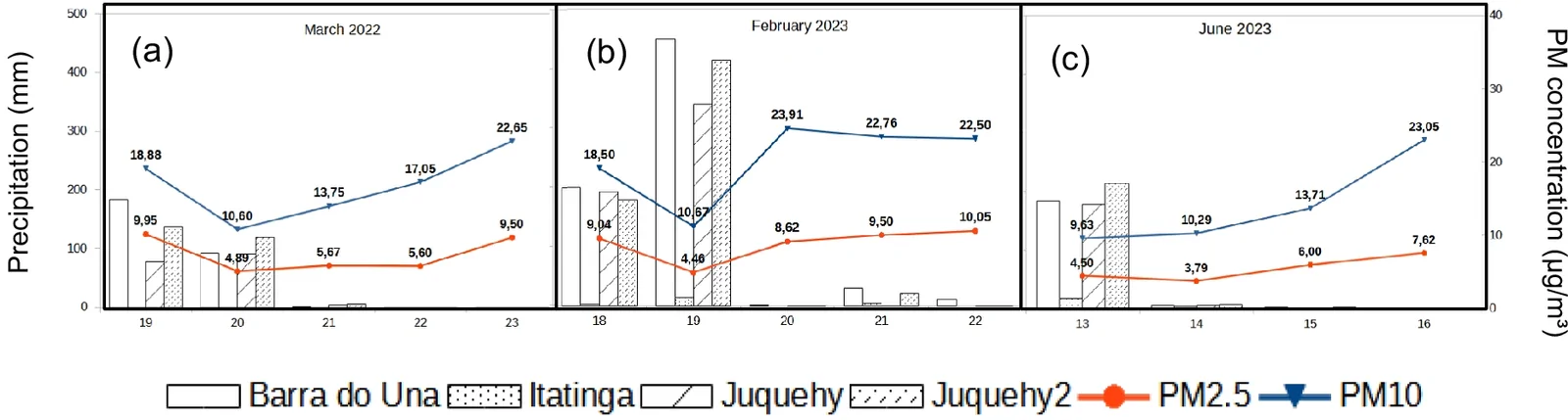
Daily precipitation (mm) at the São Sebastião rain stations and PM concentrations (μg/m^3^) in **a** March 2022, **b** February 2023, and **c** June 2023

Figure 16 compares daily precipitation and PM_10_ and PM_2.5_ concentrations during the February 2023 event with two comparative high precipitation periods. A decrease in both PM fractions was observed during periods of intense rainfall in all three cases, consistent with the wet scavenging effect of precipitation reported in previous studies (Liu et al., 2020; McMullen et al., 2021; Wang et al., 2023; Zheng et al., 2019). However, the post-rainfall behavior differed among the events.

In March 2022, PM_10_ and PM_2.5_ decreased during the high-precipitation period and subsequently increased as rainfall diminished, despite no landslides being identified in the available records. A gradual recovery was also observed after the June 2023 event, which was identified in Cluster 3 as meteorologically similar to the February event. However, PM concentrations remained below their pre-rainfall levels during the period analyzed. In contrast, following the February 2023 event, both PM fractions increased rapidly and remained elevated during the subsequent days. The unusual meteorological conditions associated with the February event, including persistent moisture supply and the influence of the Serra do Mar on rainfall enhancement, were also described by Marengo et al. (2024).

These results show that an increase in PM after intense rainfall is not restricted to events involving landslides. PM concentrations can recover over different timescales after rainfall, depending on particle size and rainfall characteristics (McMullen et al., 2021; Zheng et al., 2019). Even so, the faster and more persistent increase observed after the February 2023 event suggests that the end of rainfall or the occurrence of landslides alone cannot fully explain the post-event behavior. Together with the polarPlot, timePlot, and windRose analyses, this comparison suggests that the February increase was likely influenced by a combination of meteorological conditions and changes in local activities and emissions after the disaster. However, the available data does not allow these individual contributions to be quantified.

## Conclusions

This research investigated the behavior of PM_10_ and PM_2.5_ in São Sebastião during an extreme rainfall event and landslides. The CA was effective in different meteorological regimes, enabling the isolation of a group reflective of the critical atmospheric instability conditions associated with the February 2023 event. Although precipitation acted as an important wet removal mechanism, the spatio-temporal analysis suggests that post-event PM concentrations may also have been influenced by particle resuspension, exposed soil, and other local activities associated with the landslide response.

It is important to note that the analysis underwent significant methodological changes. For example, there are gaps in the daily time series, and the location of the only air quality monitoring station is far from the landslide epicenter in Vila Sahy, approximately 30 km. These factors may have reduced the detection of concentration peaks associated with the event. Therefore, the measurements from the monitoring station may not fully represent air quality conditions in the most severely affected areas.

This concern is reinforced by climate projections for the region: Alcântara et al. (2025a) showed that, under more severe IPCC scenarios, landslide susceptibility in São Sebastião is expected to increase due to higher projected precipitation intensities, while Veloso et al. (2025) reported that the municipality has already faced recurrent mass-movement disasters across consecutive rainy seasons. If landslides of this magnitude become more frequent, as these projections suggest, the potential post-event air quality effects associated with particle resuspension in this study may also become more frequent, reinforcing the need for adequate monitoring infrastructure in vulnerable coastal areas.

The results indicate that extreme climate events can temporarily impact air quality in coastal regions with complex topographies, in addition to their physical and social effects. The observed increase in PM concentrations following the event may be associated with PM resuspension, although this remains a hypothesis and other factors related to the landslide, such as increased traffic from rescue operations, the circulation of trucks and vehicles, and pollutant transport from nearby activities, may also have contributed. Future studies using denser monitoring networks, closer stations to affected areas, and continuous datasets may help improve our understanding of how long these effects persist.

## Data Availability

All data analyzed during this study will be available upon request.
